# On Grid-Generated Quantum Turbulence

**DOI:** 10.3390/e27101054

**Published:** 2025-10-10

**Authors:** Ladislav Skrbek

**Affiliations:** Faculty of Mathematics and Physics, Charles University, Ke Karlovu 3, 121 16 Prague, Czech Republic; ladislav.skrbek@matfyz.cuni.cz

**Keywords:** grid turbulence, helium, quantized vortices, quantum turbulence, two-fluid model

## Abstract

Nearly homogeneous and isotropic turbulence, generated in flows through grids of various forms in wind tunnels or by towing or oscillating grids in stationary samples of classical viscous fluids and the superfluid phases of helium, have played an essential role in studies of the still partly unresolved problem of turbulence in fluids. This review describes a selected class of complementary grid experiments performed with classical viscous fluids such as air or water and with the superfluid liquid phases of ^4^He (He II) and ^3^He-B, which led to a deeper understanding of the underlying physics of turbulent quantum flows. In particular, we discuss the pioneering experiments on generating and probing quantum turbulence by oscillating grids in He II in the zero temperature limit, performed by Peter McClintock’s group in Lancaster.

## 1. Introduction

Grid-generated turbulence in classical viscous fluids represents what is the current best approximation of an idealized model of turbulence—homogeneous isotropic turbulence (HIT). It can be held in a statistically steady state by balancing the forcing effects and dissipation, in which the flow properties fluctuate about well-defined mean values, or one may study its temporal decay. In classical grid-generated turbulence, most of the relevant experimental work has occurred in wind tunnels, where the turbulence decays downstream. This technique has been developed by, among others, Comte-Bellot and Corssin [1,2], Kistler and Vrebalovich [3], Sreenivasan et al. [4], and Sinhuber et al. [5]. Classical grid-generated turbulence without a mean flow has been studied by De Silva and Fernando using an oscillating grid [6] (or, in order to improve homogeneity, by a pair of oscillating grids [7,8]) and by towing a grid through a stationary sample of viscous fluid, e.g., as conducted by van Doorn et al. [9].

From a theoretical standpoint, although many practical working models are known, a first principles general theory describing HIT and its temporal decay has not yet been developed. However, influential works such as those of Kolmogorov [10,11], Comte-Bellot and Corssin [1,2], and Saffman [12,13] outline phenomenological theories relating the forms of the 3D turbulent energy spectra to the temporal decay of turbulence. This approach was extended by Skrbek and Stalp [14] and further developed by Barenghi, Skrbek, and Sreenivasan [15] by taking into account the ideas of Eyink and Thomson [16], which included the finite size of the turbulence-generating box and intermittency and viscosity effects. Recent theoretical progress in this topic is represented by the work of Migdal [17], who derived an analytic formula for the energy spectrum and dissipation of turbulence in a finite system and claims that the theoretical results obtained are in agreement with the experimental classics of Comte-Bellot and Corssin [1,2]. The title of Migdal’s paper “Quantum solution of classical turbulence: Decaying energy spectrum” suggests that quantum turbulence might play an important role in a deeper understanding of the problem at hand.

In this review, we describe grid-generated turbulence in quantum fluids, whose properties cannot be described by Navier–Stokes equations but depend on quantum mechanics. We consider the liquid superfluid phases of ^4^He (He II) and ^3^He-B [18], where grid-generated quantum turbulence [15] has been studied experimentally. Most physical properties of superfluid ^4^He and ^3^He-B are understood within the phenomenological two-fluid model [19], with the following main features. For temperatures $T<T_{\lambda }\approx 2.2$ K, liquid ^4^He, for historical reasons known as He II, is described as if composed of two interpenetrating constituents, superfluid of density $\rho_{s}$ and normal fluid of density $\rho_{n}$. For ^3^He-B, the corresponding critical temperature is $T_{c}\approx \left(1-3\right)$ mK, depending on the pressure. The viscous normal fluid consists of a gas of thermal excitations and carries the entire entropy content of the liquid. Above T≈1 K in He II and T>200μK in ^3^He-B, their mean-free-path is small and the thermal excitations can be described hydrodynamically, i.e., as a fluid with finite viscosity. The total density ρ of the liquid is nearly temperature independent and satisfies $\rho =\rho_{n}+\rho_{s}$. In the zero temperature limit, helium is composed entirely of superfluid ($\rho_{s}/\rho \to 1$ and $\rho_{n}/\rho \to 0$), while superfluidity vanishes at $T_{\lambda }$ (He II) and $T_{c}$ (^3^He-B); ($\rho_{s}/\rho \to 0$ and $\rho_{n}/\rho \to 1$). Under isothermal conditions, the two fluids move independently when flow velocities are small. When a certain critical velocity is exceeded, however, thin vortex lines are formed in the superfluid component. Their circulation is quantized [20,21] in units of $\kappa =h/M_{s}$ (usually singly), where *h* is Planck’s constant and $M_{s}$ is the mass of the superfluid particle (in He II which is bosonic, $M_{s}=m_{4}$, the mass of a ^4^He atom, while in the fermionic ^3^He-B $M_{s}=2m_{3}$, the mass of a Cooper pair, twice the mass of a ^3^He atom [22]).

In the experimentally challenging case of the zero temperature limit with no normal fluid, quantum turbulence consists only of the tangle of quantized vortex lines and can be called pure superfluid turbulence. A useful quantity characterizing its intensity is the vortex line density, *L*, which is the length of vortex line per unit volume. Two distinct and well-defined turbulent regimes exist, called Vinen (or ultra-quantum) turbulence and Kolmogorov (or quasi-classical) turbulence. In He II at finite temperatures, quantum turbulence may or may not involve turbulent motion of the normal fluid of low kinematic viscosity $\nu_{n}$, while in ^3^He-B the rather thick normal fluid in practice does not become turbulent. When vortex lines are present in the isothermal flow, the otherwise independent normal and superfluid velocity fields become coupled by the mutual friction force acting at all relevant length scales.

Mechanical forcing by a grid in the zero temperature limit results in a single turbulent velocity field of the superfluid component of He II or ^3^He-B. At finite temperature, e.g., by thermal forcing, the two components of He II can be made to flow (on average) with different velocities, which is the situation of counterflow. Forcing by a grid, however, results in coflows, the closest analogues to classical viscous grid-generated flows, in which normal and superfluid components move (on average) with the same mean velocity in the same direction. Although grid-generated quantum turbulence represents the closest analogy with classical grid-generated turbulence, there are important differences between the two. This is true even in the zero temperature limit, where only the superfluid velocity field exists and even more so at finite temperatures, where the presence of the normal component and the mutual friction force [23,24] significantly complicates the situation.

One important advantage in using the superfluid phases of helium as working fluids to study turbulence is that, in addition to detection methods which are widely used to study turbulent flows of classical viscous fluids, there is a variety of specific experimental methods that can be used to probe quantum flows, such as second sound attenuation [25,26], Andreev reflection [27,28,29] or the utilization of charged or neutral helium ions as tracers [30,31]. On the other hand, a crucial factor for the experimental investigation of quantum turbulence, especially at very low temperatures, is the purity of the investigated samples of superfluid helium. As for He II, impurities other than ^3^He can be removed relatively easily by freezing them out, but at temperatures well below 1 K, even tiny concentration of ^3^He atoms might affect the observed properties significantly. To this end, McClintock’s group in Lancaster developed a purification cryostat capable of producing ^4^He of excellent isotopic purity [32,33], suitable for such experiments.

The article is composed as follows: Following this short introduction, we describe various experimental setups used to study grid-generated quantum turbulence. We then discuss its properties, first in the zero temperature limit and then at finite temperature, where superfluid helium displays the two-fluid behavior. We then consider grid-generated quantum turbulence under rotation and finish with a short conclusion.

## 2. Experimental Setups to Study Grid-Generated Quantum Turbulence in Helium Superfluids

### 2.1. Oscillating Grids

**Figure 1:**
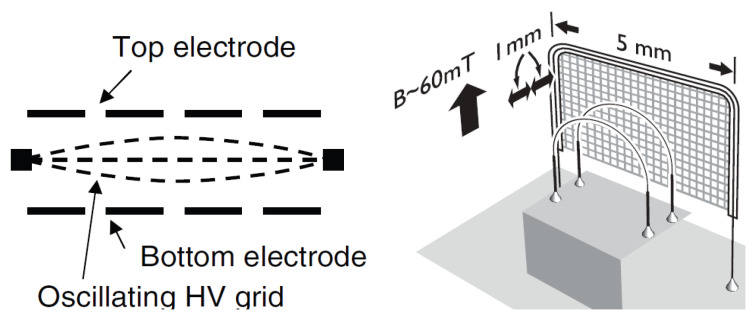
(**Left**): Schematic diagram of an electrostatically driven oscillating grid in He II. (**Right**): The arrangement of the oscillating grid and associated vorticity detector wires used to study quantum turbulence in ^3^He-B. Reproduced with permission from Nichol et al. [35] and Bradley et al. [36].

Generation of quantum turbulence in He II via an oscillating grid and its detection were first attempted in McClintock’s group in Lancaster [34]. The grid, held at a high constant electrical potential, was stretched over a round holder placed between the top and bottom flat electrodes with several holes, completing the double capacitor; see the left panel of Figure 1. Oscillations of the grid of a mesh size of about 100 μm were excited via application of an alternating potential to the lower electrode, producing quantized vorticity and, eventually, quantum turbulence above a certain threshold. With the use of negative ions (a negative ion in liquid helium represents an electron in otherwise empty bubble of hydrodynamic mass above 200 He atoms and radius 1.7 nm at the saturated vapor pressure, which is determined by a balance between the zero-point energy of the electron and the surface energy of the bubble) as a tool to probe the generated quantum flow, this pioneering experiment clearly demonstrated the production and decay of quantum turbulence in the zero-temperature limit, although only qualitatively.

In order to achieve better homogeneity of produced quantum turbulence, a pair of grids oscillating in phase, driven in He II by a room-temperature motor via a vertical shaft, have been used by Svancara and La Mantia [37] in Prague and very recently by Bret et al. [38] in Grenoble. These flows have been studied by the particle tracking velocimetry technique and second sound attenuation (second sound is an entropy, or temperature wave propagating in He II with velocity typically 20 m/s).

As for ^3^He-B, a series of vibrating grid experiments have been performed by Bradley et al. [36,39,40,41,42], utilizing the high-technology, Lancaster-style nuclear cooling stage. The grid, made of a fine mesh of copper wires spaced 50 μm apart, was attached to a vibrating wire (see the right panel of Figure 1), operated at sub mK temperatures in magnetic field at the resonance frequency of this structure, typically around 1 kHz. The generated quantum turbulence was probed by two vibrating wire resonators made from 2.5 mm diameter loops of 4.5 μm NbTi wire, positioned at distances of 1mm and 2mm from the grid. The detection of steady and decaying quantum turbulence utilized the unique method of Andreev reflection [29].

### 2.2. Towed Grids

Experiments on decaying quantum turbulence in He II generated by a towed grid have been performed over many years in Donnelly’s group at the University of Oregon [14,43,44,45]. Quantum turbulence is generated by towing a grid through a stationary sample of He II in a channel of square cross-section, about 30 cm long and 1 cm wide. Two different grids have been used over time. The first was of unconventional design—a 65% open brass monoplanar grid of rectangular tines, 1.5 mm thick, with a mesh size *M* (tine spacing) of 0.167 cm. The second, consisting of 28 rectangular tines of width 0.012 cm forming 13×13 full meshes of approximate dimension M=0.064 cm, was identical in design to that of Comte-Bellot and Corrsin [1]. The grid was attached to a stainless steel pulling rod that exited the cryostat, attached to a computer-controlled linear servo motor that positioned the grid with an accuracy of about 1mm and provided towing velocities up to $u_{G}=2.5$ m/s. This arrangement enabled the exploration of a wide range of mesh Reynolds numbers up to ReM=uGMρ/μ≤2×105, where μ is the dynamic viscosity of the normal fluid and ρ the total density of He II.

Similar towed grid experiments in both liquid He I and in He II have been performed at the University of Florida in Gainesville by Yang and Ihas [46]. The detection technique in these towed grid experiments was second sound attenuation, revealing either steady-state or decaying vortex line density in the superfluid component of He II.

More recently, towed grid experiments have been performed by Wei Guo and coworkers at Florida University [47]. Additionally to the second sound detection method, the generated decaying quantum turbulence in He II was probed by using the neutral ${\mathrm{He}}_{2}^{*}$ triplet molecules, which can be produced in liquid helium following the ionization or excitation of ground state helium atoms. They form bubbles in liquid helium with a radius of about 6 Å and can be used as tracer particles. To image them, a sophisticated laser-induced fluorescence technique was developed—a laser pulse excites them from their triplet ground state to the excited electronic d-state; most of them quickly decay to an intermediate state, emitting detectable fluorescent photons.

An interesting update of the ion detection technique combined with a towed grid in He II down to the mK range has been developed in Manchester by Zmeev et al. [48]. The device was mounted in a rotating ^3^He–^4^He dilution refrigerator and was capable of working in He II down to almost the zero-temperature limit. Additionally, the assembly could be rotated about the vertical axis, allowing accurate calibration against the well-known vortex line density in a rotating bucket of He II.

### 2.3. Superfluid Wind Tunnels

The first superfluid wind tunnel was constructed by Craig and Pellam [49] in 1957. It was of cylindrical geometry, restricted by two superleaks that allowed only a thermally generated net superflow. The authors used a propeller with mica wings and demonstrated the existence of perfect potential flow for subcritical flow velocity by observing a no lift region.

Subsequently, a number of cryogenic wind tunnels have been constructed, operating on the same principles as their complementary classical wind tunnels at ambient temperature, allowing the exploration of ^4^He as a working fluid, both for classical cryogenic flows of He I (or other working fluids) and for quantum flows of He II. All of them, however, operate at finite temperature above 1 K, where He II displays two-fluid behavior. It is very difficult to arrange a steady flow of He II (or ^3^He-B) in the zero temperature limit; such a wind tunnel represents an experimental challenge, which will alow direct comparison of classical and purely superfluid working fluids. Helium wind tunnels operating at finite temperature, driving cryogenic helium by a centrifugal pump, powered via a shaft from the room temperature flange of the cryostat, have been constructed and successfully operated by Roche and coworkers in Grenoble [50,51,52].

Another possibility, utilized in Prague, is to use cryogenic compressible bellows driving a cryogenic helium wind with a grid added downstream, see [15] and references therein. It allows investigation of both the steady-state quantum turbulence and its temporal decay.

## 3. Grid-Generated Quantum Turbulence in Zero Temperature Limit

Within the widely accepted two-fluid model, both He II and ^3^He-B are purely superfluid in the low temperature limit. Therefore, as in classical turbulence, one has to consider only one superfluid velocity field. The essential difference is, however, that this velocity field is subject to the quantum mechanical constraint that the vorticity is zero everywhere except within the cores of quantized vortices. This has serious consequences.

In classical grid-generated turbulence, we have three important length scales: the size of the turbulent box *D*, the mesh size of the grid M≤D at which the turbulent energy per unit mass *W* is supplied with the rate ε=−dW/dt, and the dissipation or Kolmogorov scale $\eta ={\left(\nu^{3}/\varepsilon \right)}^{1/4}$, where ν is the kinematic viscosity of the working fluid. The Kolmogorov scale is a statistical quantity; its characteristic feature is that here the scale-dependent Reynolds number reaches unity. Neglecting intermittency (but see later), the so called Richardson cascade operates and, thanks to the advection and stretching of eddies of various sizes, an inertial range of scales forms between *M* and η, where the spectral energy density depends on the wave number *k* via the famous Kolmogorov roll-off exponent: $\phi \left(k\right)=C_{K}\varepsilon^{2/3}k^{-5/3}$. Here $C_{K}$ denotes the Kolmogorov constant, experimentally determined as $C_{K}=1.62\pm 0.17$ [53]. According to present understanding, the low *k* part of the spectrum acquires a $k^{2}$ slope by the equipartition theorem, in agreement with the Birkhoff–Saffman invariant. When the energy input at *M* (where the energy-containing eddies have been created) is stopped, the energy-containing scale grows with time and could saturate at *D*. If considerable spacing between *D* and η persists, the temporal decay of turbulent energy follows the form $W\propto t^{-2}$ at long times. Thanks to the relation $\varepsilon =\nu \langle \omega^{2}\rangle$ (where $\langle \omega^{2}\rangle$ means the mean square vorticity) that follows from the Navier–Stokes equation, this transforms to the law $\omega \left(t\right)\propto t^{-3/2}$ for decaying vorticity at long times. We note that these decay laws remain unchanged in the limit of vanishing viscosity.

In reality, things are more complicated [54] than Kolmogorov in 1941 (K41) assumed, namely that small enough scales *ℓ* can be considered homogeneous and isotropic, with universal statistics that depend on the inertial interval of scales only through one relevant parameter: ε. If it were so, then the velocity of size *ℓ* eddies may be estimated as ${\left(\varepsilon \ell \right)}^{1/3}$ and velocity structure functions $S_{n}\left(R\right)$, should scale as ${\left(\varepsilon \ell \right)}^{n/3}$. In practice, however, accurate experiments and numerical simulations show that scaling exponents $\zeta_{n}$ deviate from the K41 prediction $\zeta_{n}^{K41}=n/3$. In 1962 Kolmogorov assumed Gaussian statistics of ln[ε(ℓ)] resulting in the phenomenological K62 log-normal model $\zeta_{n}^{K62}=n/3-\mu_{I}n\left(n-3\right)/18$, where $\mu_{I}$ is the intermittency correction. The various approaches to estimation of the intermittency corrections are, however, beyond the scope of this paper.

In pure superfluid HIT, contrary to classical HIT, there is no viscosity and, hence, no dissipative scale. The flow exists down to the smallest scales—sizes of the vortex cores $\xi_{4}\approx 1$ Å in He II and $\xi_{3}\approx 10-60\;$nm, depending on pressure, in ^3^He-B. Analogous with classical Reynolds number (which, for inviscid superfluid, cannot be defined), we define the superfluid Reynolds number by replacing ν by κ of the same dimension and introduce the length scale $\ell_{Q}={\left(\kappa^{3}/\varepsilon \right)}^{1/4}$ called the quantum length scale. It marks the transition between (i) quasiclassical scales where the quantization of circulation or the “granularity” of superfluid turbulence does not matter and (ii) the quantum scales for which quantum restrictions are essential. The condition that the superfluid Reynolds number at $\ell_{Q}$ is of order unity requires that $\ell_{Q}$ is equal to a mean distance between quantized vortices in the tangle, i.e., $\ell_{Q}\approx L^{-1/2}$. We emphasize that the very existence of $\ell_{Q}$ is the direct consequence of quantum mechanics, as in the quasiclassical limit of vanishing Planck’s constant $\ell_{Q}\to 0$. The Richardson cascade, based on the advection and stretching of turbulent eddies of various sizes, can operate at large quasiclassical scales (i), where classical-like eddies exist thanks to partial polarization of the vortex tangle but cannot proceed beyond $\ell_{Q}$ (ii), as due to the Kelvin theorem, an individual quantized vortex cannot be stretched. On the other hand, no viscous dissipation mechanism operates around $\ell_{Q}$. The transfer of turbulent energy farther down the smaller scales is still possible, however, via a different mechanism mediated by the Kelvin wave cascade on individual vortex lines and reconnections (possibly with formation of a bottleneck around $\ell_{Q}$) until a dissipation mechanism—phonon emission by Kelvin waves in He II or the excitation of Caroli–Matricon states in vortex cores in ^3^He-B—becomes effective. It can be approximated by introduction of the effective kinematic viscosity of turbulent superfluid (of order 0.1κ) and the corresponding effective dissipation scale $\eta_{Q}$.

In pure superfluid HIT, we have to consider one more important length scale, $\ell_{Q}$, which intervenes in the following way: For large $\ell_{Q}>M$ the Richardson cascade cannot operate but turbulent energy can still be transferred down the scales, via the Kelvin wave cascade and reconnections. This type of quantum turbulence is called Vinen turbulence. The vortex tangle is approximately random and the temporal decay of vortex line density is of the form $L\propto t^{-1}$ as first discussed (in the context of thermal counterflow) by Vinen [55]. Upon increasing the energy input ε, $\ell_{Q}$ becomes smaller than *M*, the classical-like Richardson cascade starts to operate, and an inertial range of scales is established between these scales. This is the Kolmogorov type of quantum turbulence, containing large vortex structures which can be thought of as composed of vortex-line bundles, achieved by partial polarization of the tangle. It displays quasiclassical decay, i.e., the vortex line density decays as $L\left(t\right)\propto t^{-3/2}$. At scales smaller than $\ell_{Q}$, the quantum turbulence is, however, always of the Vinen type. Transition from Vinen to Kolmogorov type of quantum turbulence upon increasing the energy input ε at the scale *M* in the zero temperature limit has been observed in ^3^He-B Lancaster experiments with an oscillating grid [36,42,56]. It follows that, even in the simplest case of the zero temperature limit, grid-generated pure superfluid turbulence is more complex than its classical counterpart.

In fact, determination of the effective kinematic viscosity $\nu_{\mathrm{eff}}$ in the zero temperature limit is not straightforward. In He II, by towing a grid through superfluid helium, Zmeev et al. [48] produced probably the best realization of quasiclassical quantum HIT filling a channel. By using the ion technique, the Manchester group measured the temporal decay rate of vortex line density, which followed the classical decay of vorticity in HIT. Following Vinen [57], assuming that in pure superfluid Kolmogorov turbulence the energy flux can be expressed as $\varepsilon =\nu_{\mathrm{eff}}\kappa^{2}L^{2}$, the quasiclassical decay law $L\left(t\right)\propto t^{-3/2}$ can be used to estimate $\nu_{\mathrm{eff}}\approx 0.1\;\kappa$. This value is, however, about an order of magnitude higher than that estimated from the decay of quantum turbulence generated by an impulsive spin-down to rest of a cubic cell. It is believed that this discrepancy arises due to the change of the effective boundary conditions from no-slip to slip because of the loss of traction at the container walls below about 0.8 K. Similar behavior was observed in Helsinki by Eltsov et al. [58] in ^3^He-B.

It is interesting to compare various aspects of steady-state classical and pure superfluid turbulence (i.e., quantum turbulence in the zero temperature limit) generated by oscillating grids. The classical case can be characterized as follows [6]: The rms fluid velocity at a distance *x* from the grid with mesh *M* and the integral length scale $\ell_{0}$ follow the experimentally established laws:

(1) $$u_{\mathrm{rms}}=C_{1}s^{3/2}M^{1/2}f/x;\;\;\;\ell_{0}=C_{3}x$$

where $C_{1}$ and $C_{3}$ are numerical factors that depend on the details of a particular grid (typically $C_{1}\approx 0.3;\;C_{3}\approx 0.12$, *s* is the stroke of the grid and *f* is the frequency of the grid oscillations. Strictly speaking, the grid turbulence generated in this way is not isotropic. The distance *x* is measured from a virtual origin that is slightly displaced from the grid position by a distance of order *M*. The integral length scale $\ell_{0}$ is closely related to the size of the energy-containing eddies. Application of these relationships leads to the conclusion that significant turbulent intensity cannot penetrate to a depth as large as 1.5 mm from the Lancaster grid, despite it being detected there experimentally [40]. It follows that the classical scenario is not applicable and that the steady-state quantum turbulence generated in the T→0 limit in ^3^He-B by an oscillating grid differs from its classical counterpart.

### Transition to Grid-Generated Quantum Turbulence in Zero Temperature Limit

In this limit the transition occurs from inviscid (i.e., potential) flow of the single superfluid component and, as the normal fluid is absent, it is the simplest case to consider. The transition is unique, without a classical counterpart and it has to be discussed in connection with the vortex nucleation problem in superfluids. A detailed discussion of critical velocities and vortex nucleation (see, e.g., [39,59,60,61] and general discussion of transition to quantum turbulence is beyond the scope of this article. Here we limit the discussion by stating that one has to distinguish between the intrinsic (nucleation of a quantum vortex in a vortex-free sample) and extrinsic (nucleation originating from already present seeds) cases. In practice, in He II extrinsic nucleation almost always occurs, while in ^3^He-B both intrinsic and extrinsic nucleation are possible.

The transition to grid-generated quantum turbulence in the zero temperature limit in both He II and ^3^He-B has been investigated experimentally in a series of experiments in Lancaster. In both these superfluids oscillating grids were used that were, however, of different types.

In ^3^He-B, the peak velocity u of the grid attached to an oscillating wire shown in the right of Figure 1 [39] increased linearly with the applied driving force *F*, which was interpreted as due to internal damping of the oscillatory structure; the same as in vacuum. Around u≈1 mm/s this dependence changed character to $u\propto \sqrt{F}$, typical of the turbulent drag force in classical turbulence. We note in passing that the authors of [39] did not report any change in resonant frequency.

In the following Lancaster ^3^He-B experiment of Bradley et al. [40] with the oscillating grid the quantum turbulence produced was detected using the technique of Andreev reflection, sensed by a vibrating wire resonator nearby. Its fractional decrease in damping was converted into the vortex line density *L* produced by the oscillating grid. With this technique, using the vibration amplitude of the grid above a certain value, it was possible to observe the transition to QT. While the detailed processes are no doubt complex, the current view is that the vibrating grid emits vortex rings more frequently with increasing grid velocity. At some critical velocity the density of rings becomes sufficiently high that they can no longer avoid each other. Subsequent reconnection brings more complex shapes and a cascade of further reconnections leading rapidly to quantum turbulence. This scenario was backed by Tsubota’s Osaka group simulations [41].

Another Lancaster ^3^He-B experiment of Bradley et al. with an oscillating grid [36] demonstrated the cross-over from the Vinen to Kolmogorov forms of quantum turbulence. By increasing the grid forcing, more and more energy was supplied to the flow at the mesh scale *M*, all of which being dissipated in the steady state. Bradley et al. [36] investigated the temporal decay of vortex line density originating from various levels of intensity of the steady state. The late temporal decay changed its form from $L\propto t^{-1}$ (signature of Vinen type of quantum turbulence) to $L\propto t^{-3/2}$ (signature of Kologorov type of quantum turbulence) in a bounded domain. In a subsequent complementary experiment Bradley et al. [42] measured directly the energy decay rate ε of grid-generated turbulence inside a small box acting as a black body radiator of quasiparticles. The late-time decay changed in character, from $\varepsilon \propto t^{-2}$ to $\varepsilon =\nu_{\mathrm{eff}}\kappa^{2}L^{2}\propto t^{-3}$; consequently, the turbulent energy decayed as $W\propto t^{-2}$, in agreement with the late decay of turbulent energy in classical 3D HIT in a bounded domain. It is remarkable that, leaving aside caveats such as homogeneity and isotropy of the turbulence inside the black box radiator, the decay of pure superfluid turbulence was found to be surprisingly similar to the known decay of classical 3D HIT. The results also confirm that the key phenomenological relationship, $\varepsilon =-dW/dt=\nu_{\mathrm{eff}}{\left(\kappa L\right)}^{2}$, first suggested by Vinen [57], is meaningful for the Kolmogorov type of pure superfluid turbulence.

**Figure 2:**
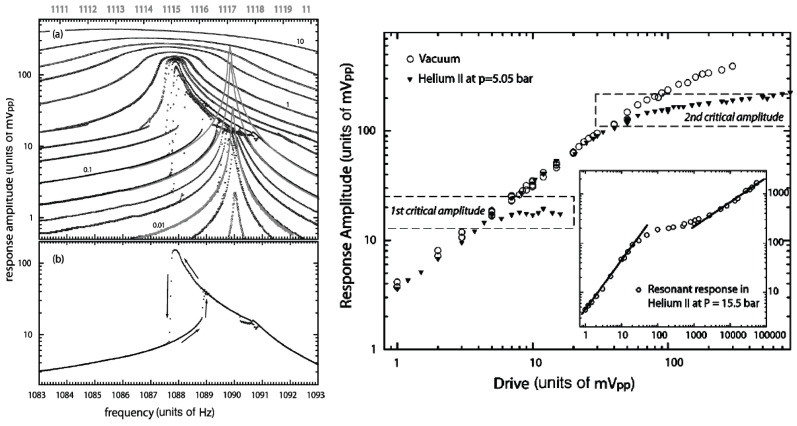
Left: (**a**) Resonance curves measured with the lock-in amplifier at 5.05 bar for drive levels (in V_*pp*_) of 0.001, 0.005, 0.01, 0.02, 0.03, 0.05, 0.1, 0.2, 0.3, 0.5, 1, 2, 3, 5, and 10. Each drive level is represented by two separate curves recorded for frequency sweeps in opposite directions. There is an intermediate range of driving levels where hysteresis loops with two stable branches arise but, otherwise, the two sweeps produce identical results. The superimposed (almost) Lorentzian resonances (smooth, gray-scale) represent the responses to 0.01, 0.05, and 0.1 V_*pp*_ drives in vacuum (upper frequency axis; the frequency shift is due to the hydrodynamic added mass of the grid oscillating in He II). (**b**) For clarity, a separate plot of the data for the 0.2 V_*pp*_ drive is shown as an example of results recorded at intermediate drive amplitude. The arrows indicate direction around the hysteresis loop. Right: Response amplitude of the grid versus the drive level at resonance measured in vacuum (open symbols) and in He II at p = 5.05 bar (main figure). The positions of the first and the second thresholds are indicated in order to emphasize that above the first threshold the damping remains unchanged, provided that the measurements are recorded in sequence from high drive toward low drive. The inset shows the drive dependence of the response amplitude of the grid at p = 15.5 bar, indicating regimes of laminar and turbulent flow. The full lines indicate the linear and square-root responses. Reproduced with permission from Nichol et al. [35].

In He II, the Lancaster oscillating grid experiments performed in the group of McClintock with the large grid shown in the left of Figure 1 revealed complex results [35,62], illustrated in Figure 2. As shown in the left, with growing amplitude of oscillation, the flow changed character at a first critical threshold from pure inviscid superflow to a flow regime that was believed to involve a boundary layer composed of quantized vortex loops. The oscillatory motion of the grid acquired highly nonlinear features, including double-valued (reentrant) resonance curves (one such a hysteretic loop is shown for clarity in the bottom left panel) and a decrease in the resonant frequency with increasing drive amplitude, but for a certain drive interval without any appreciable increase in damping. Upon further increasing the drive level, a second critical threshold was attained, where the resonant frequency reached a nearly stable value, the response amplitude almost stopped growing, but the linewidth increased. As shown in the inset in the right panel of Figure 2, upon further increase of the drive level the response amplitude acquired a square-root drive dependence.

In order to explain the existence of two thresholds Vinen with coworkers [63] proposed the following scenario: They assumed that the first critical velocity, connected to a frequency shift rather than changes in the drag on the oscillating grid, is associated with seeds of quantized vortex loops pinned to the rough surface of the grid, possibly forming a thin boundary layer. Indeed, the response of a single vortex loop of length *b* can be described via normal modes of excited Kelvin waves (If a vortex line is deformed into a helix, the deformation propagates as a wave, as discussed for the classical case by Lord Kelvin [64], who derived its dispersion relation consisting of two branches $\omega^{\pm }$. It holds for a quantum vortex with quantized circulation κ; for He II vortices the slow branch $\omega^{-}$ is relevant. In approximation of long wavelength 2π/k and hollow core it reads $\omega^{-}≅\left(\kappa k^{2}/4\pi \right)\left[\ln\left(1/ka\right)-0.116\right]$, where a≅0.15 nm is the vortex core parameter. For further details, see [65]) with wave numbers $k_{n}=n/b$. This response will be adiabatic (at any instant the vortex is at its equilibrium position) if the oscillation frequency is significantly lower than that of the fundamental resonant Kelvin mode. The superflow however distorts the vortices. The impulse required to create a vortex loop is $\rho_{s}\kappa S$, where *S* is the area of vortex loop, which oscillates in an oscillatory flow. For such a pinned vortex loop, we can write the rate of change of the momentum of the oscillating grid of bare mass $M_{0}$ as

(2) $$M_{\mathrm{eff}}\frac{du}{dt}=M_{0}\frac{du}{dt}+M_{\mathrm{enh}}\frac{du}{dt}+\rho_{s}\kappa \frac{dS_{\mathrm{pr}}}{dt}\;,$$

where $M_{\mathrm{enh}}$ is the hydrodynamically enhanced mass of the grid and $S_{\mathrm{pr}}$ is the change of the loop area projected in the direction perpendicular to superflow. If the vortex responds adiabatically, we can write $dS_{\mathrm{pr}}/dt=\left(dS_{\mathrm{pr}}/du\right)\left(du/dt\right)$, so that $M_{\mathrm{eff}}=M_{0}+M_{\mathrm{enh}}+\rho_{s}\kappa dS_{\mathrm{pr}}/du$. Although the quantity $dS_{\mathrm{pr}}/du$ must be found by appropriate simulations for a given configuration, this simple approach illustrates the mechanism of how the effective mass of the oscillating structure increases, leading to the shift in the resonant frequency of the flow due to an oscillating grid, with no appreciable increase in dissipation. An initial vortex loop of arbitrary orientation tends to twist in the oscillatory superflow and eventually self-reconnects upon exceeding a second critical threshold. These vortex loops carry energy and momentum from the grid and propagate into the bulk, eventually merging in a random turbulent tangle, creating most likely the Vinen type of quantum turbulence, i.e., the scenario discussed above for the case of oscillating grid in ^3^He-B.

This, however, might not be the end of the story. We can take advantage of the known results from the Prague/Lancaster experiments with oscillating tuning forks [66], where yet another, third threshold occurs upon further increase of the drive. In this case the second threshold is usually accompanied by hysteresis (detectable with amplitude sweeps) and an increase in the drag, though the measured drag coefficient is only of the order $10^{-1}$–$10^{-2}$. It, therefore, differs significantly from classical turbulent flows (where the drag coefficients are close to unity) by the absence of large vortical structures in the wake. It is only upon exceeding the third threshold, critical velocity, observed to be of the order of 1 m/s and above, that the drag coefficient starts to grow toward unity even in the zero temperature limit. This happens when a turbulent wake is created at length scales exceeding the intervortex distance, mimicking the classical turbulent wake, representing quasi-classical or Kolmogorov quantum turbulence.

In fact, this scenario with three critical thresholds is in qualitative agreement with the results just discussed from the oscillating grid in He II as illustrated in Figure 2. Considering the inset in the right panel, we see that the first threshold, occurring at the drive level slightly below 0.01 V_*pp*_, is not visible, being buried in the linear drive dependence shown in the inset (there is no drag offered by He II to the grid in this linear regime; the observed linear dependence is shown to be the same as measured in vacuum due to intrinsic damping caused by deformation of the grid material). It shows, however, the second threshold, around 0.03 V_*pp*_, where the drag coefficient steeply grows, indicating the transition to quantum turbulence. In analogy with ^3^He-B, it is most likely in the form of Vinen quantum turbulence. Finally, after reaching the third threshold, at the drive levels above 1 V_*pp*_, the drag coefficient becomes constant (Kolmogorov quantum turbulence), i.e., it acquires the form typical of turbulent drag in classical turbulence. We emphasize that a possibility of this scenario is the consequence of the interplay between the injection (mesh) and the quantum length scales in quantum turbulence.

It is desirable to check whether this rather speculative scenario is observable upon increasing the drive in experiments with other suitably designed oscillating structures in He II and perhaps represents the general scenario for transition to quantum turbulence in any flow of He II, besides its flows due to oscillating objects. Unfortunately, it seems experimentally impossible to check an analogy of this scenario in ^3^He-B, as the cooling power of cryostats at submilli-Kelvin temperatures is insufficient to compensate for such a strong energy dissipation.

## 4. Grid-Generated Quantum Turbulence in Two-Fluid Regime

At sufficiently high temperatures, both ^3^He-B and He II display two-fluid behavior. Unfortunately, the grid generated turbulence in ^3^He-B at these temperatures cannot be effectively studied experimentally. The reason is that, due to the high viscosity of the normal fluid, ^3^He-B flow through the grid results in intolerable heating. With regard to the two-fluid model, we therefore discuss the grid turbulence in He II at temperatures above 1 K, where the mean free path of rotons and phonons is short enough to allow the classical hydrodynamic two-fluid description. When forced by a grid, both the superfluid and normal components easily become turbulent. The grid turbulence therefore represents a unique double turbulent state, consisting of a continuum of normal fluid eddies and tangles of quantized vortices. There are two turbulent velocity fields, interacting with each other via the mutual friction force. Although the double turbulent state is, in general, very complex, under some conditions, remarkable similarities with classical grid turbulence exist. Above 1 K in He II we have to examine both the superfluid and the normal fluid energy spectra. Isothermal incompressible flow of the normal fluid of He II can be described using the Navier–Stokes equations with density $\rho_{n}$ and very low kinematic viscosity $\eta_{n}$ of order κ. It is characterized by the analogue of the Reynolds number in classical viscous fluids: the Donnelly number, which is defined as $\mathrm{Dn}=RU/\nu_{n}$, where *R* and *U* are the characteristic length scale and velocity and $\nu_{n}$ stands for the kinematic viscosity of the normal fluid alone, $\nu_{n}=\mu /\rho_{n}$.

Let us consider the example of grid turbulence in He II flow driven at mesh scale $M\gg \ell_{Q}$ at temperature slightly below 1.5 K, where $\nu_{n}\approx \kappa$. For simplicity, let us assume that the mutual friction is switched off. The Donnelly number is equal to the superfluid Reynolds number, Dn=Res, and $\ell_{Q}$ in the superfluid is equal to the Kolmogorov dissipation scale $\eta_{n}$ in the normal fluid. At scales sufficiently larger than $\ell_{Q}$ or $\eta_{n}$, the turbulent spectra in both fluids are naturally matched. However, while in the normal fluid the Richardson cascade is terminated at $\eta_{n}$, in the superfluid it continues via Kelvin wave cascade along individual vortex lines until dissipation occurs in the form of phonon radiation as described above. Let the mutual friction now be switched on. By departing from the temperature where $\nu_{n}\approx \kappa$, $\ell_{Q}$ and $\eta_{n}$ are no longer matched. Still, not much happens at large enough scales. Upon approaching smaller and smaller scales, however, the matching is gradually lost and dissipation due to mutual friction starts to operate (resulting in the roll-off exponent becoming gradually steeper), and one component starts to act as a source or drain for the other.

**Figure 3:**
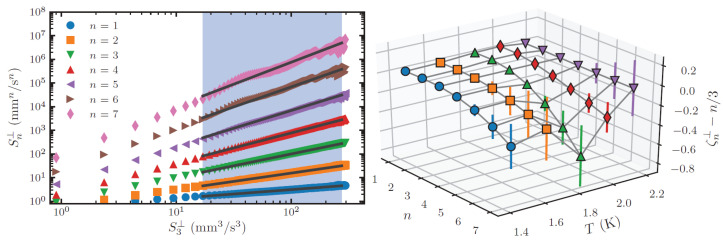
(**Left**): Extended self-similarity. Transverse velocity structure functions $S_{n}$ for *n* = 1–7, plotted using the extended similarity hypothesis versus $S_{3}$. The particular case shown is for 1.85 K, 300 mm/s grid velocity, and 4 s decay time. Other cases appear qualitatively similar. (**Right**): Intermittency corrections to the scaling exponents of the transverse structure functions deduced through extended self-similarity for data obtained at 4 s decay time and with grid velocity $v_{g}=300$ mm/s. The three-dimensional plot shows the temperature-dependent deviation of scaling exponents from the Kolmogorov–Obukhov scaling: 1.45 K, 1.65 K, 1.85 K, 2.00 K, and 2.15 K. Reproduced with permission from Varga et al. [69].

This results in an increase of intermittency corrections [54], as predicted by Boue et al. [67] and Biferale et al. [68], experimentally confirmed by Varga et al. [69]. The experiment utilized the Tallahassee tracer-line visualization setup [70]. A mesh grid of 7 × 7 woven wires was towed by a linear motor to move past flow probes, producing nearly homogeneous and isotropic turbulence. To probe it, besides a standard second-sound attenuation method revealing temporal decay of vortex-line density L(t), high-intensity femtosecond laser pulses were sent through the channel, producing a thin line of H$e_{2}^{*}$ molecular tracers, completely entrained by the viscous normal fluid with negligible effect from the superfluid or quantized vortices [31]. A line of the molecules so created was then left to evolve for a drift time $t_{d}$ of about 10–30 ms before it was visualized by laser-induced fluorescence using a separate laser sheet at 905 nm for imaging. The streamwise velocity v(x) can be determined by dividing the displacement of a line segment at *x* by $t_{d}$ and used to evaluate the transverse velocity increments needed for structure function calculations. They are defined as $S_{n}^{⊥}\left(r\right)=\langle {\left|v\left(x\right)-v\left(x+r\right)\right|}^{n}\rangle$, where *v* is the transverse turbulent velocity with respect to the direction r. Intermittency corrections are estimated by statistical analysis of the experimental data, via higher-order structure functions $S_{n}\left(r\right)$ that are sensitive to occurrence of rare events. In order for $S_{n}^{⊥}$ to be evaluated accurately, the experimental estimation of the probability density functions of transverse velocity need to have well-resolved tails and requires therefore large data sets. Fortunately, even though the errors on the directly evaluated structure functions are significant, by using the extended self-similarity hypothesis (ESS) [71], the structure function scaling exponents $\zeta_{n}^{⊥}$ are clearly displayed (see examples in Figure 3) and can be determined fairly accurately, from a linear fit to $\log S_{n}^{⊥}$ vs. $\log S_{3}^{⊥}$. This is precisely what the authors have achieved. The right panel of Figure 3 shows the deduced scaling exponents $\zeta_{n}^{⊥}$, for all investigated temperatures, as a function of the order *n*. It is remarkable that the deduced intermittency corrections closely follow the theoretical prediction of Biferale et al. [68] that the maximum of the intermittency correction occurs around T≈1.95 K, where the normal and superfluid densities are matched.

The double turbulent state of quantum turbulence in He II is therefore a unique turbulent system, where intermittency can be tuned simply by setting the temperature. This is in contrast to classical turbulence in single-component viscous fluids, where many different classical turbulent (nearly HIT) flows display almost the same intermittency corrections [72]. It will be interesting to improve experimental resolution, allowing reliably extend the scaling range beyond n=7, in order to verify the recent numerical prediction of Buaria and Sreenivasan [73] that the transverse Lagrangian scaling exponents saturate at ≈2 for n≥8 in quantum grid-generated turbulence.

On the other hand, measurements of Rusaouen et al. [74], performed not in grid-generated quantum turbulence but in the wake of a disk, found no appreciable temperature dependence in intermittency corrections. This appears to be controversial, however, there are reasons for different results. First, the prediction of temperature-dependent enhanced intermittency is explained by the authors of Refs. [67,68] via a flip-flop scenario, a random energy transfer between the normal and superfluid components due to mutual friction. While H$e_{2}^{*}$ molecules probe the normal fluid solely, the cantilever anemometer and pressure probes used in the Rusaouen et al. experiment [74] may not sense such a flip-flop exchange of energy, as they probe both fluids simultaneously. Secondly, the sizes of these probes are typically larger than the quantum length scale $\ell_{Q}$, where the characteristics of classical and quantum turbulence are essentially the same.

Furthermore, from the theoretical/numerical standpoint, is fair to mention that very recently Polanco et al. [75] put results on temperature dependence of intermittency corrections under scrutiny, based on direct numerical simulation of coarse-grained Hall–Vinen–Bekharevich–Khalatnikov (HVBK) model [15]. Their claims are at variance with, e.g., those of Biferale et al. [68], suggesting a need to revisit the delicate problem of intermittency in quantum turbulence in further dedicated experimental, theoretical and numerical studies.

### Kolmogorov 4/5-Law in Grid-Generated Quantum Turbulence

Often cited as the only exact result of classical fully developed turbulence is the so-called Kolmogorov 4/5-law [10]. It states that, within the inertial range of scales, the third-order longitudinal velocity structure function is given by $S_{3}^{\left|\right|}\left(r\right)=\left(-4/5\right)\varepsilon r$. Its validity in grid-generated quantum turbulence was tested in Grenoble by Salort et al. [52] in their He II wind tunnel. The authors used the experimental fact [51] that, keeping the same mean-flow velocity above and below the transition temperature $T_{\lambda }$, ε does not change. In order to estimate it, they used He I velocity recordings, since He I is a classical fluid, where the Kolmogorov 4/5-law is known to be valid and used that value to compensate the third-order velocity structure function obtained in He II. In this way, they observed a plateau for nearly half a decade of scales, corresponding to the resolved inertial range of the turbulent cascade. This is the first experimental evidence that the 4/5-law is valid in He II, which was further backed up by high-resolution simulations.

## 5. Grid-Generated Quantum Turbulence Under Rotation

It is well known that, under rotation, classical 3D turbulence acquires two-dimensional (2D) features. As an example, let consider the temporal decay of energy per unit mass of the grid generated nearly HIT which, without rotation (past initial transition stage), is well described by a power law $E\left(t\right)=u^{2}\left(t\right)\propto {\left(t-t_{\mathrm{vo}}\right)}^{-n}$, where *n* is the decay exponent and $t_{\mathrm{vo}}$ is a virtual origin. The value of the exponent *n* depends on whether the size of the energy containing eddies is free to grow (n≈6/5) or is bounded by the domain size (n≈2). For decaying vorticity ω this transfers, using the relationship $\varepsilon =-dE/dt=\nu \omega^{2}$, to n≈11/10 and n≈3/2 [14].

Morize and Moisy [76] studied the energy decay of grid-generated turbulence in a rotating tank by means of particle image velocimetry. They used a water filled glass tank of square section, 35 cm on the side, mounted on a rotating turntable, whose angular velocity Ω was varied between 0.13 and 4.34 rad/s. After the fluid was set in solid body rotation, turbulence was generated by rapidly towing a co-rotating square grid of square bars of 1 cm with a mesh size of M=39 mm. The authors discussed three characteristic times: in addition to the instantaneous turnover time M/u, where $M\approx \ell_{\mathrm{ece}}$ is the characteristic size of the energy-containing eddies, two other time scales are present in the problem, which have opposite effects on the turbulence decay. One is the rotation time scale $T_{\mathrm{rot}}=\Omega^{-1}$ and, for bounded systems, there is also the Ekman time scale, $T_{\mathrm{Ek}}=h{\left(\nu \Omega \right)}^{-1/2}$, where *h* is the characteristic size along the rotation axis. The rotation time scale is associated with the propagation of inertial waves, which modify the nonlinear energy transfers and reduce the energy dissipation, an effect which results in a lower value of the decay exponent *n*. The Ekman time scale governs the dissipation of those inertial waves from multiple reflections in the Ekman layers, thus enhancing the energy decay at large time, and shortening the range for a possible self-similar decay even at large Reynolds numbers. Experimentally, clear evidence of the reduction of the energy decay by the rotation has been observed for times smaller than the Ekman time scale, although due to complex interplay between global quantities, like the grid Rossby number or the Ekman time scale and more local quantities, a detailed description of the decay of confined rotating turbulence is a rather delicate issue.

There is a clear call to investigate rotating grid-generated quantum turbulence, especially in the zero temperature limit, which represents the intellectually simplest but experimentally most difficult case. It represents the complementary flow allowing direct measurements of quantities which are either impossible or at least very difficult to measure in classical grid turbulence. The primary example is vortex line density, directly related to vorticity in classical grid turbulence. It will, however, be a challenging task experimentally. To probe such a quantum flow, helium ions or excimer molecules can be utilized in He II, while in ^3^He-B Andreev reflection can be used. It should be compared with the two-fluid He II case above 1 K, which can be probed by second sound attenuation. In both cases the measurable quantity will be the vortex line density, *L*, which can be related to vorticity as ω=κL. Additionally, it is interesting to test the existence of an analogy of Ekman layer and its possible role in the decay of grid-generated quantum turbulence, in particular at very low temperatures, where the no-slip boundary condition at the wall of the vessel ceases to hold.

In fact, a similar experiment in He II has already been recently performed in Prague [77], with rotating turbulent thermal counterflow, probed by second sound attenuation. The late time decay exponent for vortex line density indeed decreases with the rotation rate; from 1.5 at rest to about 0.75 at $\Omega \approx 1\;s^{-1}$ and the rotating vertical counterflow acquires 2D features. It is not surprising, as two-dimensionalization of rapidly rotating turbulence is a well-established property of classical turbulence [78]. On the other hand, as steady thermal counterflow of He II does not have a classical analogue, direct comparison with rotating grid generated turbulence of viscous fluids is not justified. Dedicated experimental investigation of various aspects of rotating grid-generated quantum turbulence is desired to clarify this interesting issue.

## 6. Conclusions

Quantum turbulence, in common with classical turbulence in viscous fluids, comes in many forms. In this review, classical 3D nearly homogeneous and isotropic grid-generated turbulence has served as the basis for the description of grid-generated turbulence in two quantum fluids: superfluid He II and ^3^He-B. We chose these working fluids for the following reasons. First, laboratory samples of these two quantum fluids are in most cases macroscopically large, allowing agitation of a large number of degrees of freedom by grids of various form, similarly to the classical case. This enables direct comparison between grid-generated turbulence in classical fluids and in quantum fluids. Secondly, this comparison is strengthened by the fact that some of the same methods of study can be applied to both the classical and quantum cases. Third, some detection methods from low temperature physics research are unique to quantum turbulence, providing additional quantities which cannot be measured (or only with great difficulty) in classical grid turbulence.

Of great importance here is the intellectually simplest but experimentally challenging case of the zero-temperature limit which has no viscosity and where the consequence of quantized vorticity in superfluid can be studied directly. New elements emerge, such as the new quantum length scale $\ell_{Q}$, reflecting the severe quantum mechanical restriction that a superflow is potential everywhere except inside vortex cores. A new form of turbulence, Vinen-type, has been identified, alongside the quasi-classical Kolmogorov-type, which more closely resembles classical fluid turbulence. We have proposed a three-step scenario describing the transition to quantum turbulence in flows due to grids or other structures oscillating in He II at very low temperature. In the absence of viscous dissipation, new sinks of turbulent energy have been discovered, namely dissipative mutual friction, phonon radiation, and the excitation of Caroli–Matricon bound states in the vortex cores. Despite the similarities to classical grid turbulence, pure superfluid grid-generated turbulence is different and more complex than the classical case.

Various studies of grid-generated quantum turbulence in the zero temperature limit, since the pioneering experiment with the oscillating grid of the group of McClintock, have brought interesting results. While some findings are firmly established, serious gaps are still to be filled, such as clarifying the role of rotation on grid-generated quantum turbulence and its temporal decay. We hope that this review will stimulate further dedicated investigations, widening our understanding of the underlying physics of turbulence in fluids.

## Data Availability

No new data were created or analyzed in this study.
